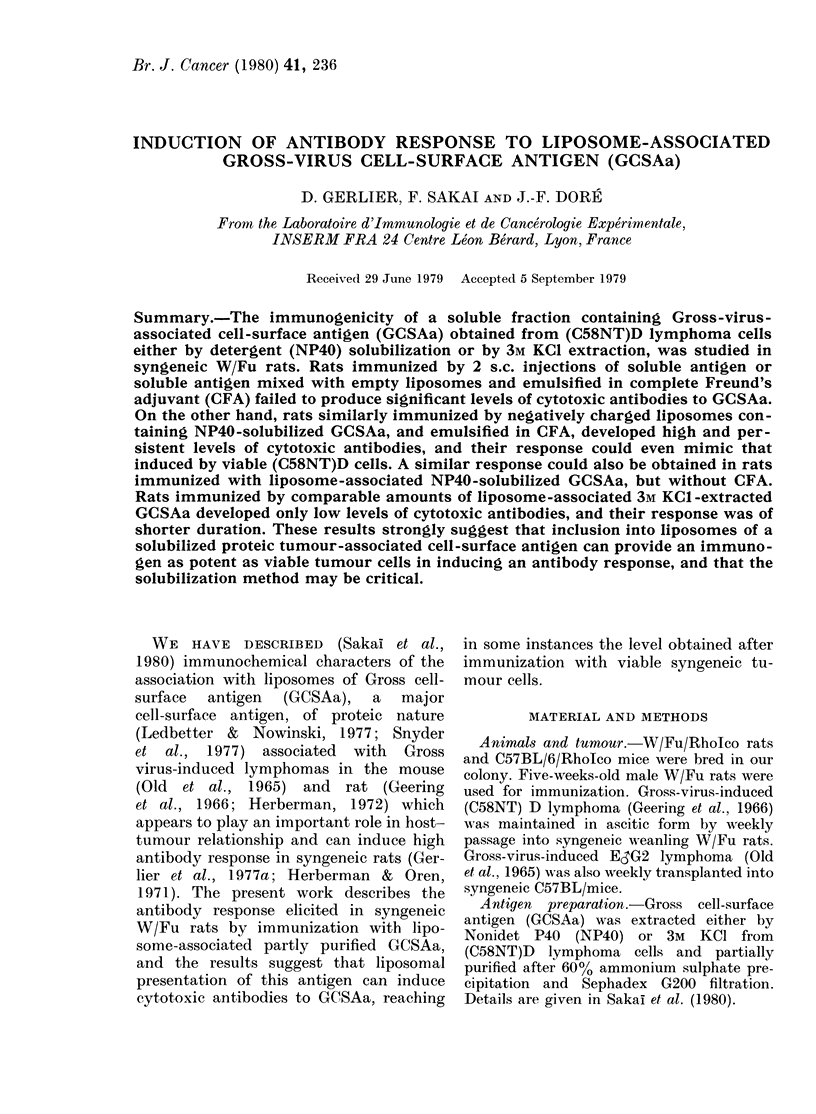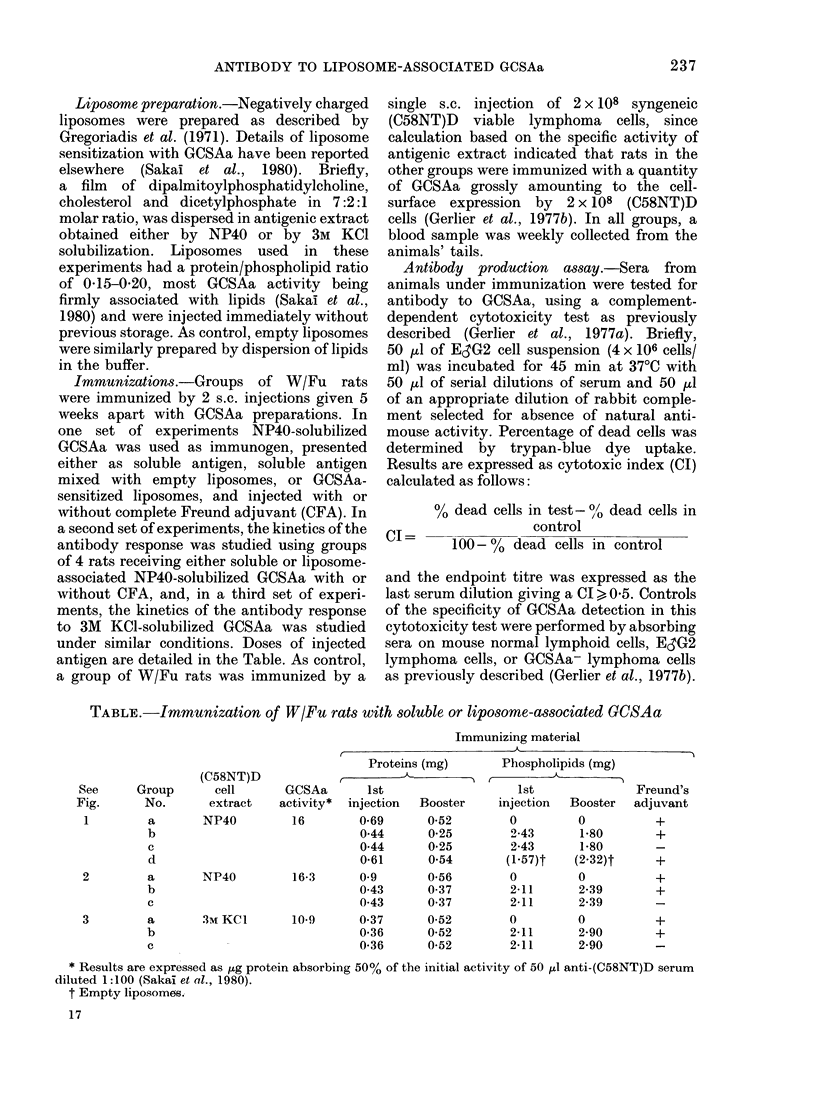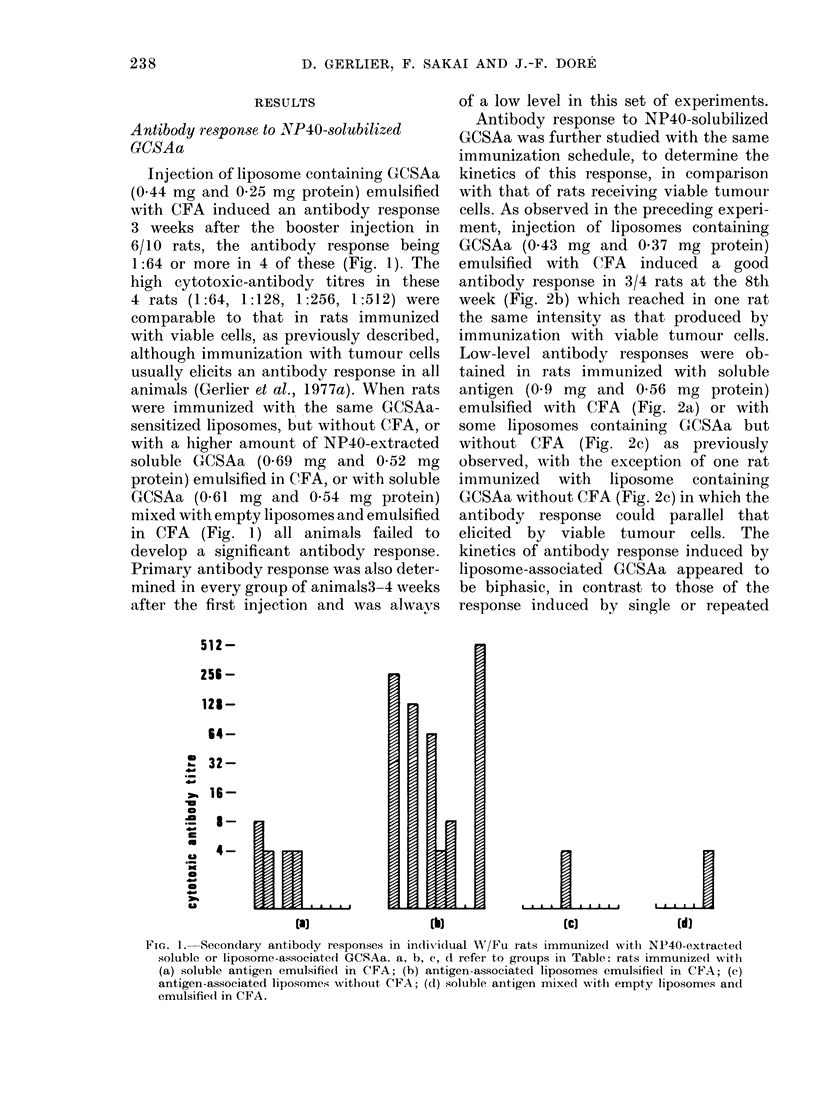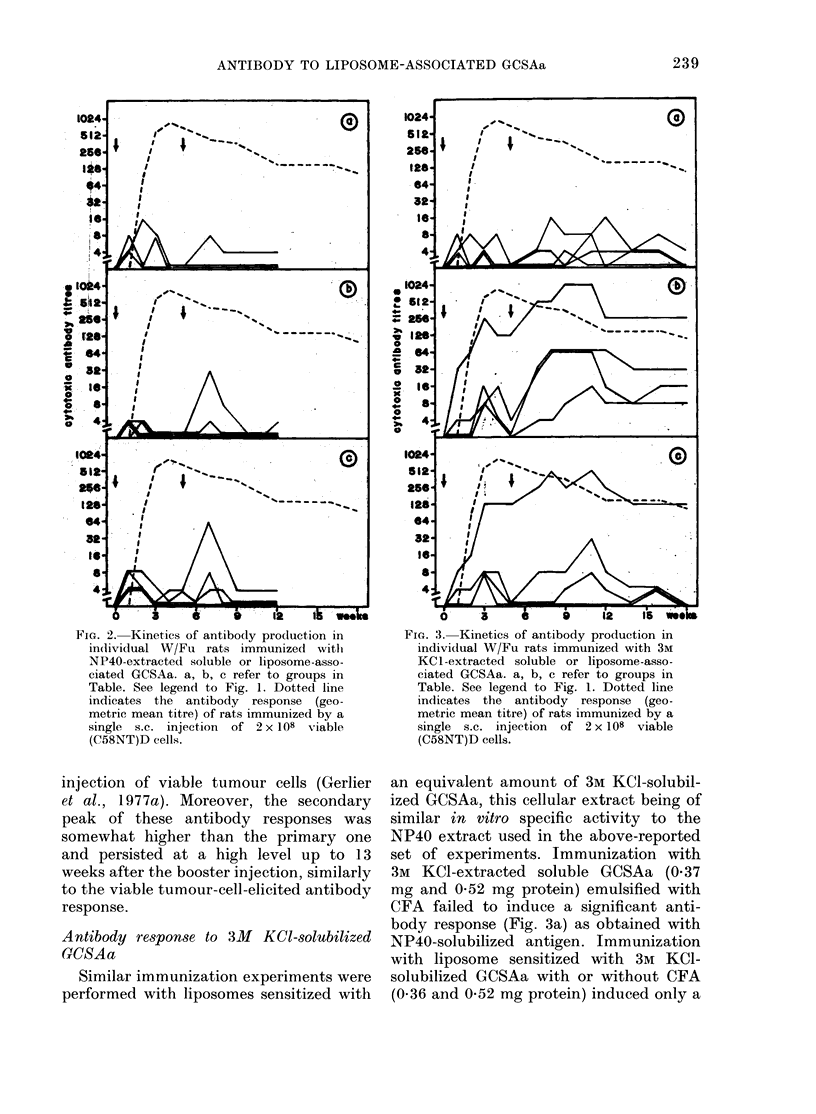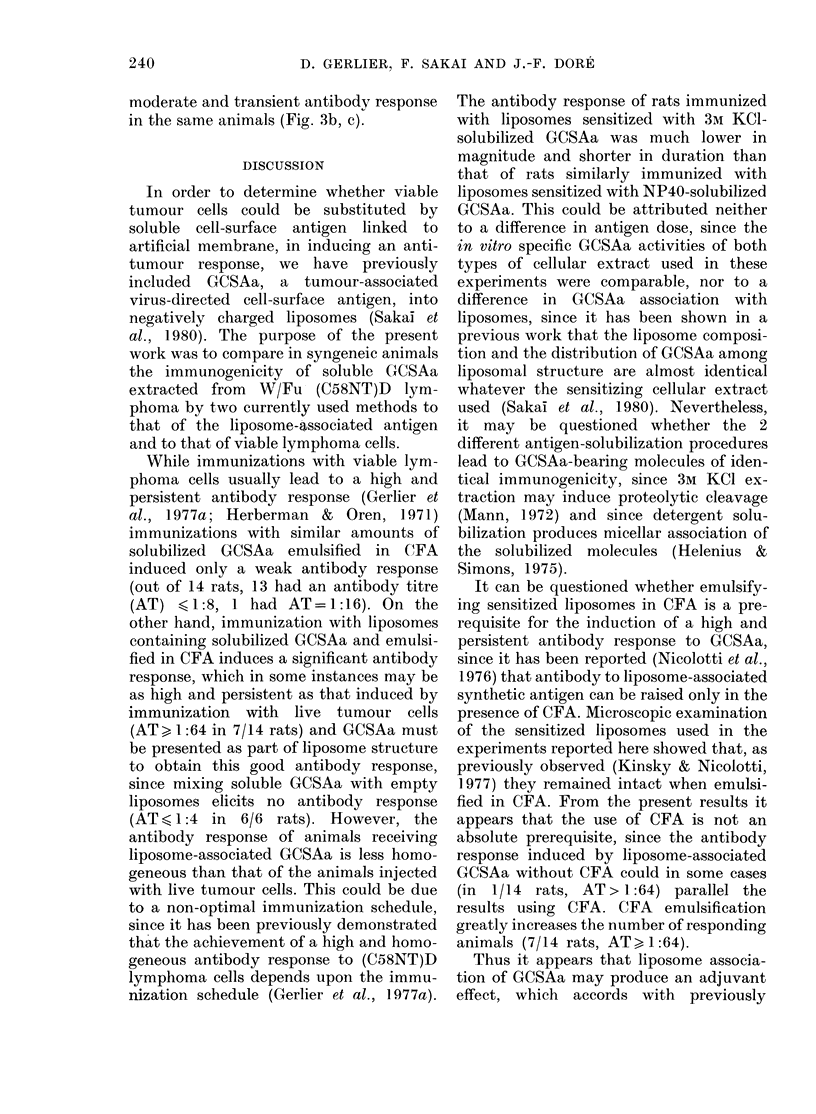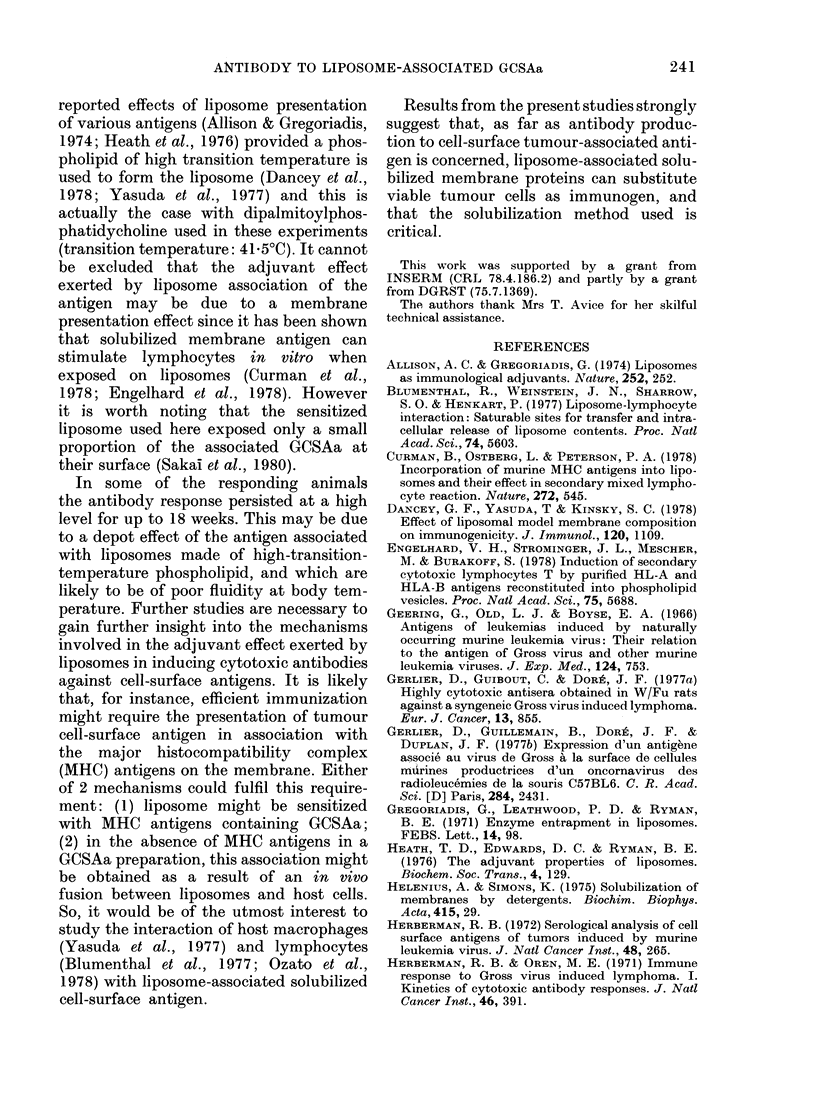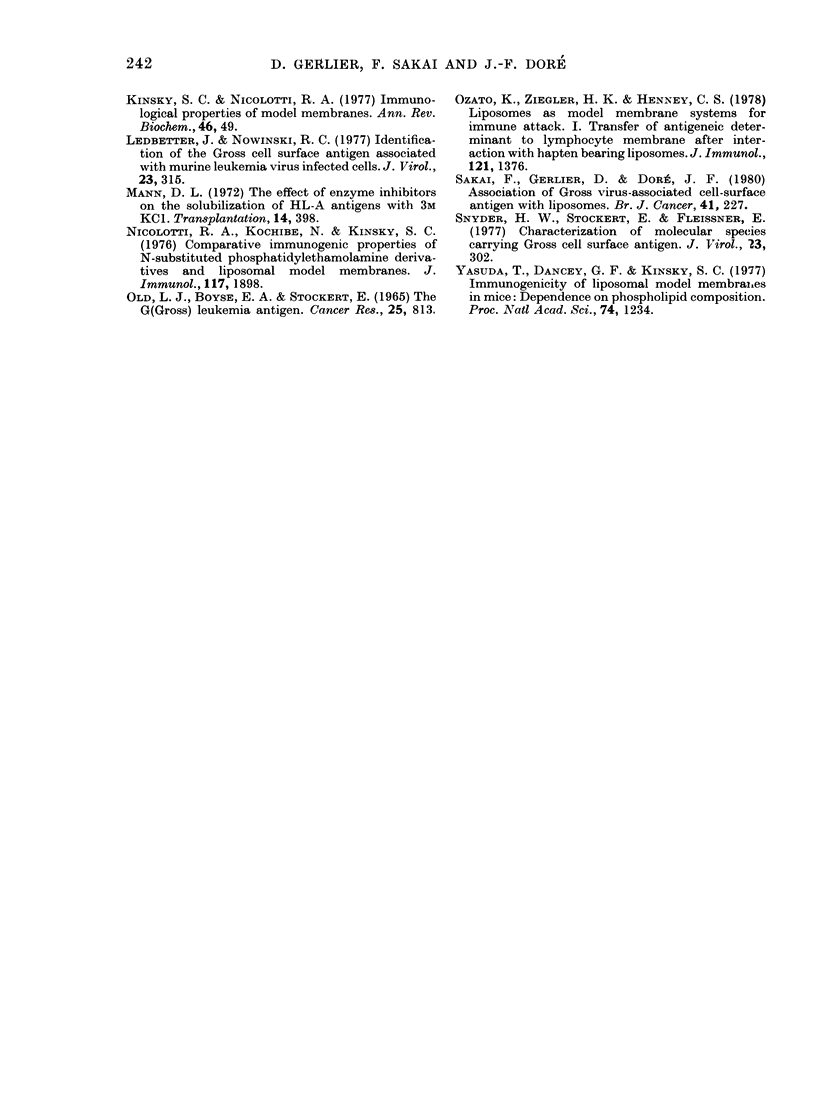# Induction of antibody response to liposome-associated Gross-virus cell-surface antigen (GCSAa).

**DOI:** 10.1038/bjc.1980.35

**Published:** 1980-02

**Authors:** D. Gerlier, F. Sakai, J. F. Doré

## Abstract

The immunogenicity of a soluble fraction containing Gross-virus-associated cell-surface antigen (GCSAa) obtained from (C58NT)D lymphoma cells either by detergent (NP40) solubilization or by 3M KCl extraction, was studied in syngeneic W/Fu rats. Rats immunized by 2 s.c. injections of soluble antigen or soluble antigen mixed with empty liposomes and emulsified in complete Freund's adjuvant (CFA) failed to produce significant levels of cytotoxic antibodies to GCSAa. On the other hand, rats similarly immunized by negatively charged liposomes containing NP40-solubilized GCSAa, and emulsified in CFA, developed high and persistent levels of cytotoxic antibodies, and their response could even mimic that induced by viable (C58NT)D cells. A similar response could also be obtained in rats immunized with liposome-associated NP40-solubilized GCSAa, but without CFA. Rats immunized by comparable amounts of liposome-assocated 3M KCl-extracted GCSAa developed only low levels of cytotoxic antibodies, and their response was of shorter duration. These results strongly suggest that inclusion into liposomes of a solubilized proteic tumour-associated cell-surface antigen can provide an immunogen as potent as viable tumour cells in inducing an antibody response, and that the solubilization method may be critical.


*Br. J. Cancer* (1980) **41**, 236

### INDUCTION OF ANTIBODY RESPONSE TO LIPOSOME-ASSOCIATED GROSS-VIRUS CELL-SURFACE ANTIGEN (GCSAa)

D. GERLIER, F. SAKAI AND J.-F. DORÉ

*From the Laboratoire d'Immunologie et de Cancérologie Expérimentale, INSERM FRA 24 Centre Léon Bérard, Lyon, France*

Received 29 June 1979 Accepted 5 September 1979

**Summary.—The immunogenicity of a soluble fraction containing Gross-virus-associated cell-surface antigen (GCSAa) obtained from (C58NT)D lymphoma cells either by detergent (NP40) solubilization or by 3M KCl extraction, was studied in syngeneic W/Fu rats. Rats immunized by 2 s.c. injections of soluble antigen or soluble antigen mixed with empty liposomes and emulsified in complete Freund's adjuvant (CFA) failed to produce significant levels of cytotoxic antibodies to GCSAa. On the other hand, rats similarly immunized by negatively charged liposomes containing NP40-solubilized GCSAa, and emulsified in CFA, developed high and persistent levels of cytotoxic antibodies, and their response could even mimic that induced by viable (C58NT)D cells. A similar response could also be obtained in rats immunized with liposome-associated NP40-solubilized GCSAa, but without CFA. Rats immunized by comparable amounts of liposome-associated 3M KCl-extracted GCSAa developed only low levels of cytotoxic antibodies, and their response was of shorter duration. These results strongly suggest that inclusion into liposomes of a solubilized proteic tumour-associated cell-surface antigen can provide an immunogen as potent as viable tumour cells in inducing an antibody response, and that the solubilization method may be critical.**

WE HAVE DESCRIBED (Sakaï *et al.*, 1980) immunochemical characters of the association with liposomes of Gross cell-surface antigen (GCSAa), a major cell-surface antigen, of proteic nature (Ledbetter & Nowinski, 1977; Snyder *et al.*, 1977) associated with Gross virus-induced lymphomas in the mouse (Old *et al.*, 1965) and rat (Geering *et al.*, 1966; Herberman, 1972) which appears to play an important role in host–tumour relationship and can induce high antibody response in syngeneic rats (Gerlier *et al.*, 1977*a*; Herberman & Oren, 1971). The present work describes the antibody response elicited in syngeneic W/Fu rats by immunization with liposome-associated partly purified GCSAa, and the results suggest that liposomal presentation of this antigen can induce cytotoxic antibodies to GCSAa, reaching in some instances the level obtained after immunization with viable syngeneic tumour cells.

### MATERIAL AND METHODS

*Animals and tumour.*—W/Fu/RhoIco rats and C57BL/6/RhoIco mice were bred in our colony. Five-weeks-old male W/Fu rats were used for immunization. Gross-virus-induced (C58NT) D lymphoma (Geering *et al.*, 1966) was maintained in ascitic form by weekly passage into syngeneic weanling W/Fu rats. Gross-virus-induced E♂G2 lymphoma (Old *et al.*, 1965) was also weekly transplanted into syngeneic C57BL/mice.

*Antigen preparation.*—Gross cell-surface antigen (GCSAa) was extracted either by Nonidet P40 (NP40) or 3M KCl from (C58NT)D lymphoma cells and partially purified after 60% ammonium sulphate precipitation and Sephadex G200 filtration. Details are given in Sakaï *et al.* (1980).

*Liposome preparation.*—Negatively charged liposomes were prepared as described by Gregoriadis *et al.* (1971). Details of liposome sensitization with GCSAa have been reported elsewhere (Sakaï *et al.*, 1980). Briefly, a film of dipalmitoylphosphatidylcholine, cholesterol and dicetylphosphate in 7:2:1 molar ratio, was dispersed in antigenic extract obtained either by NP40 or by 3M KCl solubilization. Liposomes used in these experiments had a protein/phospholipid ratio of 0·15–0·20, most GCSAa activity being firmly associated with lipids (Sakaï *et al.*, 1980) and were injected immediately without previous storage. As control, empty liposomes were similarly prepared by dispersion of lipids in the buffer.

*Immunizations.*—Groups of W/Fu rats were immunized by 2 s.c. injections given 5 weeks apart with GCSAa preparations. In one set of experiments NP40-solubilized GCSAa was used as immunogen, presented either as soluble antigen, soluble antigen mixed with empty liposomes, or GCSAa-sensitized liposomes, and injected with or without complete Freund adjuvant (CFA). In a second set of experiments, the kinetics of the antibody response was studied using groups of 4 rats receiving either soluble or liposome-associated NP40-solubilized GCSAa with or without CFA, and, in a third set of experiments, the kinetics of the antibody response to 3M KCl-solubilized GCSAa was studied under similar conditions. Doses of injected antigen are detailed in the Table. As control, a group of W/Fu rats was immunized by a single s.c. injection of $2 \times 10^8$ syngeneic (C58NT)D viable lymphoma cells, since calculation based on the specific activity of antigenic extract indicated that rats in the other groups were immunized with a quantity of GCSAa grossly amounting to the cell-surface expression by $2 \times 10^8$ (C58NT)D cells (Gerlier *et al.*, 1977*b*). In all groups, a blood sample was weekly collected from the animals' tails.

*Antibody production assay.*—Sera from animals under immunization were tested for antibody to GCSAa, using a complement-dependent cytotoxicity test as previously described (Gerlier *et al.*, 1977*a*). Briefly, 50 μl of E♂G2 cell suspension ($4 \times 10^6$ cells/ml) was incubated for 45 min at 37°C with 50 μl of serial dilutions of serum and 50 μl of an appropriate dilution of rabbit complement selected for absence of natural anti-mouse activity. Percentage of dead cells was determined by trypan-blue dye uptake. Results are expressed as cytotoxic index (CI) calculated as follows:

$$CI = \frac{\%\text{ dead cells in test} - \%\text{ dead cells in control}}{100 - \%\text{ dead cells in control}}$$

and the endpoint titre was expressed as the last serum dilution giving a CI $\geq$ 0·5. Controls of the specificity of GCSAa detection in this cytotoxicity test were performed by absorbing sera on mouse normal lymphoid cells, E♂G2 lymphoma cells, or $GCSAa^-$ lymphoma cells as previously described (Gerlier *et al.*, 1977*b*).

TABLE.—*Immunization of W/Fu rats with soluble or liposome-associated GCSAa*

| | | | | Immunizing material | | | | |
|---|---|---|---|---|---|---|---|---|
| | | | | Proteins (mg) | | Phospholipids (mg) | | |
| See Fig. | Group No. | (C58NT)D cell extract | GCSAa activity* | 1st injection | Booster | 1st injection | Booster | Freund's adjuvant |
| 1 | a | NP40 | 16 | 0·69 | 0·52 | 0 | 0 | + |
| | b | | | 0·44 | 0·25 | 2·43 | 1·80 | + |
| | c | | | 0·44 | 0·25 | 2·43 | 1·80 | – |
| | d | | | 0·61 | 0·54 | (1·57)† | (2·32)† | + |
| 2 | a | NP40 | 16·3 | 0·9 | 0·56 | 0 | 0 | + |
| | b | | | 0·43 | 0·37 | 2·11 | 2·39 | + |
| | c | | | 0·43 | 0·37 | 2·11 | 2·39 | – |
| 3 | a | 3M KCl | 10·9 | 0·37 | 0·52 | 0 | 0 | + |
| | b | | | 0·36 | 0·52 | 2·11 | 2·90 | + |
| | c | | | 0·36 | 0·52 | 2·11 | 2·90 | – |

* Results are expressed as μg protein absorbing 50% of the initial activity of 50 μl anti-(C58NT)D serum diluted 1:100 (Sakaï *et al.*, 1980).

† Empty liposomes.

### RESULTS

### *Antibody response to NP40-solubilized GCSAa*

Injection of liposome containing GCSAa (0·44 mg and 0·25 mg protein) emulsified with CFA induced an antibody response 3 weeks after the booster injection in 6/10 rats, the antibody response being 1:64 or more in 4 of these (Fig. 1). The high cytotoxic-antibody titres in these 4 rats (1:64, 1:128, 1:256, 1:512) were comparable to that in rats immunized with viable cells, as previously described, although immunization with tumour cells usually elicits an antibody response in all animals (Gerlier *et al.*, 1977*a*). When rats were immunized with the same GCSAa-sensitized liposomes, but without CFA, or with a higher amount of NP40-extracted soluble GCSAa (0·69 mg and 0·52 mg protein) emulsified in CFA, or with soluble GCSAa (0·61 mg and 0·54 mg protein) mixed with empty liposomes and emulsified in CFA (Fig. 1) all animals failed to develop a significant antibody response. Primary antibody response was also determined in every group of animals3–4 weeks after the first injection and was always of a low level in this set of experiments.

Antibody response to NP40-solubilized GCSAa was further studied with the same immunization schedule, to determine the kinetics of this response, in comparison with that of rats receiving viable tumour cells. As observed in the preceding experiment, injection of liposomes containing GCSAa (0·43 mg and 0·37 mg protein) emulsified with CFA induced a good antibody response in 3/4 rats at the 8th week (Fig. 2b) which reached in one rat the same intensity as that produced by immunization with viable tumour cells. Low-level antibody responses were obtained in rats immunized with soluble antigen (0·9 mg and 0·56 mg protein) emulsified with CFA (Fig. 2a) or with some liposomes containing GCSAa but without CFA (Fig. 2c) as previously observed, with the exception of one rat immunized with liposome containing GCSAa without CFA (Fig. 2c) in which the antibody response could parallel that elicited by viable tumour cells. The kinetics of antibody response induced by liposome-associated GCSAa appeared to be biphasic, in contrast to those of the response induced by single or repeated

FIG. 1.—Secondary antibody responses in individual W/Fu rats immunized with NP40-extracted soluble or liposome-associated GCSAa. a, b, c, d refer to groups in Table: rats immunized with (a) soluble antigen emulsified in CFA; (b) antigen-associated liposomes emulsified in CFA; (c) antigen-associated liposomes without CFA; (d) soluble antigen mixed with empty liposomes and emulsified in CFA.

FIG. 2.—Kinetics of antibody production in individual W/Fu rats immunized with NP40-extracted soluble or liposome-associated GCSAa. a, b, c refer to groups in Table. See legend to Fig. 1. Dotted line indicates the antibody response (geometric mean titre) of rats immunized by a single s.c. injection of $2 \times 10^8$ viable (C58NT)D cells.

FIG. 3.—Kinetics of antibody production in individual W/Fu rats immunized with 3M KCl-extracted soluble or liposome-associated GCSAa. a, b, c refer to groups in Table. See legend to Fig. 1. Dotted line indicates the antibody response (geometric mean titre) of rats immunized by a single s.c. injection of $2 \times 10^8$ viable (C58NT)D cells.

injection of viable tumour cells (Gerlier *et al.*, 1977*a*). Moreover, the secondary peak of these antibody responses was somewhat higher than the primary one and persisted at a high level up to 13 weeks after the booster injection, similarly to the viable tumour-cell-elicited antibody response.

### *Antibody response to 3M KCl-solubilized GCSAa*

Similar immunization experiments were performed with liposomes sensitized with an equivalent amount of 3M KCl-solubilized GCSAa, this cellular extract being of similar *in vitro* specific activity to the NP40 extract used in the above-reported set of experiments. Immunization with 3M KCl-extracted soluble GCSAa (0·37 mg and 0·52 mg protein) emulsified with CFA failed to induce a significant antibody response (Fig. 3a) as obtained with NP40-solubilized antigen. Immunization with liposome sensitized with 3M KCl-solubilized GCSAa with or without CFA (0·36 and 0·52 mg protein) induced only a moderate and transient antibody response in the same animals (Fig. 3b, c).

### DISCUSSION

In order to determine whether viable tumour cells could be substituted by soluble cell-surface antigen linked to artificial membrane, in inducing an anti-tumour response, we have previously included GCSAa, a tumour-associated virus-directed cell-surface antigen, into negatively charged liposomes (Sakaī *et al.*, 1980). The purpose of the present work was to compare in syngeneic animals the immunogenicity of soluble GCSAa extracted from W/Fu (C58NT)D lymphoma by two currently used methods to that of the liposome-associated antigen and to that of viable lymphoma cells.

While immunizations with viable lymphoma cells usually lead to a high and persistent antibody response (Gerlier *et al.*, 1977*a*; Herberman & Oren, 1971) immunizations with similar amounts of solubilized GCSAa emulsified in CFA induced only a weak antibody response (out of 14 rats, 13 had an antibody titre (AT) ⩽1:8, 1 had AT=1:16). On the other hand, immunization with liposomes containing solubilized GCSAa and emulsified in CFA induces a significant antibody response, which in some instances may be as high and persistent as that induced by immunization with live tumour cells (AT⩾1:64 in 7/14 rats) and GCSAa must be presented as part of liposome structure to obtain this good antibody response, since mixing soluble GCSAa with empty liposomes elicits no antibody response (AT⩽1:4 in 6/6 rats). However, the antibody response of animals receiving liposome-associated GCSAa is less homogeneous than that of the animals injected with live tumour cells. This could be due to a non-optimal immunization schedule, since it has been previously demonstrated that the achievement of a high and homogeneous antibody response to (C58NT)D lymphoma cells depends upon the immunization schedule (Gerlier *et al.*, 1977*a*). The antibody response of rats immunized with liposomes sensitized with 3M KCl-solubilized GCSAa was much lower in magnitude and shorter in duration than that of rats similarly immunized with liposomes sensitized with NP40-solubilized GCSAa. This could be attributed neither to a difference in antigen dose, since the *in vitro* specific GCSAa activities of both types of cellular extract used in these experiments were comparable, nor to a difference in GCSAa association with liposomes, since it has been shown in a previous work that the liposome composition and the distribution of GCSAa among liposomal structure are almost identical whatever the sensitizing cellular extract used (Sakaī *et al.*, 1980). Nevertheless, it may be questioned whether the 2 different antigen-solubilization procedures lead to GCSAa-bearing molecules of identical immunogenicity, since 3M KCl extraction may induce proteolytic cleavage (Mann, 1972) and since detergent solubilization produces micellar association of the solubilized molecules (Helenius & Simons, 1975).

It can be questioned whether emulsifying sensitized liposomes in CFA is a prerequisite for the induction of a high and persistent antibody response to GCSAa, since it has been reported (Nicolotti *et al.*, 1976) that antibody to liposome-associated synthetic antigen can be raised only in the presence of CFA. Microscopic examination of the sensitized liposomes used in the experiments reported here showed that, as previously observed (Kinsky & Nicolotti, 1977) they remained intact when emulsified in CFA. From the present results it appears that the use of CFA is not an absolute prerequisite, since the antibody response induced by liposome-associated GCSAa without CFA could in some cases (in 1/14 rats, AT>1:64) parallel the results using CFA. CFA emulsification greatly increases the number of responding animals (7/14 rats, AT⩾1:64).

Thus it appears that liposome association of GCSAa may produce an adjuvant effect, which accords with previously reported effects of liposome presentation of various antigens (Allison & Gregoriadis, 1974; Heath *et al.*, 1976) provided a phospholipid of high transition temperature is used to form the liposome (Dancey *et al.*, 1978; Yasuda *et al.*, 1977) and this is actually the case with dipalmitoylphosphatidycholine used in these experiments (transition temperature: 41·5°C). It cannot be excluded that the adjuvant effect exerted by liposome association of the antigen may be due to a membrane presentation effect since it has been shown that solubilized membrane antigen can stimulate lymphocytes *in vitro* when exposed on liposomes (Curman *et al.*, 1978; Engelhard *et al.*, 1978). However it is worth noting that the sensitized liposome used here exposed only a small proportion of the associated GCSAa at their surface (Sakaï *et al.*, 1980).

In some of the responding animals the antibody response persisted at a high level for up to 18 weeks. This may be due to a depot effect of the antigen associated with liposomes made of high-transition-temperature phospholipid, and which are likely to be of poor fluidity at body temperature. Further studies are necessary to gain further insight into the mechanisms involved in the adjuvant effect exerted by liposomes in inducing cytotoxic antibodies against cell-surface antigens. It is likely that, for instance, efficient immunization might require the presentation of tumour cell-surface antigen in association with the major histocompatibility complex (MHC) antigens on the membrane. Either of 2 mechanisms could fulfil this requirement: (1) liposome might be sensitized with MHC antigens containing GCSAa; (2) in the absence of MHC antigens in a GCSAa preparation, this association might be obtained as a result of an *in vivo* fusion between liposomes and host cells. So, it would be of the utmost interest to study the interaction of host macrophages (Yasuda *et al.*, 1977) and lymphocytes (Blumenthal *et al.*, 1977; Ozato *et al.*, 1978) with liposome-associated solubilized cell-surface antigen.

Results from the present studies strongly suggest that, as far as antibody production to cell-surface tumour-associated antigen is concerned, liposome-associated solubilized membrane proteins can substitute viable tumour cells as immunogen, and that the solubilization method used is critical.

This work was supported by a grant from INSERM (CRL 78.4.186.2) and partly by a grant from DGRST (75.7.1369).

The authors thank Mrs T. Avice for her skilful technical assistance.

REFERENCES

Allison, A. C. & Gregoriadis, G. (1974) Liposomes as immunological adjuvants. *Nature*, **252**, 252.

Blumenthal, R., Weinstein, J. N., Sharrow, S. O. & Henkart, P. (1977) Liposome-lymphocyte interaction: Saturable sites for transfer and intracellular release of liposome contents. *Proc. Natl Acad. Sci.*, **74**, 5603.

Curman, B., Ostberg, L. & Peterson, P. A. (1978) Incorporation of murine MHC antigens into liposomes and their effect in secondary mixed lymphocyte reaction. *Nature*, **272**, 545.

Dancey, G. F., Yasuda, T & Kinsky, S. C. (1978) Effect of liposomal model membrane composition on immunogenicity. *J. Immunol.*, **120**, 1109.

Engelhard, V. H., Strominger, J. L., Mescher, M. & Burakoff, S. (1978) Induction of secondary cytotoxic lymphocytes T by purified HL-A and HLA-B antigens reconstituted into phospholipid vesicles. *Proc. Natl Acad. Sci.*, **75**, 5688.

Geering, G., Old, L. J. & Boyse, E. A. (1966) Antigens of leukemias induced by naturally occurring murine leukemia virus: Their relation to the antigen of Gross virus and other murine leukemia viruses. *J. Exp. Med.*, **124**, 753.

Gerlier, D., Guibout, C. & Doré, J. F. (1977*a*) Highly cytotoxic antisera obtained in W/Fu rats against a syngeneic Gross virus induced lymphoma. *Eur. J. Cancer*, **13**, 855.

Gerlier, D., Guillemain, B., Doré, J. F. & Duplan, J. F. (1977*b*) Expression d'un antigène associé au virus de Gross à la surface de cellules murines productrices d'un oncornavirus des radioleucémies de la souris C57BL6. *C. R. Acad. Sci.* [D] Paris, **284**, 2431.

Gregoriadis, G., Leathwood, P. D. & Ryman, B. E. (1971) Enzyme entrapment in liposomes. FEBS. Lett., **14**, 98.

Heath, T. D., Edwards, D. C. & Ryman, B. E. (1976) The adjuvant properties of liposomes. *Biochem. Soc. Trans.*, **4**, 129.

Helenius, A. & Simons, K. (1975) Solubilization of membranes by detergents. *Biochim. Biophys. Acta*, **415**, 29.

Herberman, R. B. (1972) Serological analysis of cell surface antigens of tumors induced by murine leukemia virus. *J. Natl Cancer Inst.*, **48**, 265.

Herberman, R. B. & Oren, M. E. (1971) Immune response to Gross virus induced lymphoma. I. Kinetics of cytotoxic antibody responses. *J. Natl Cancer Inst.*, **46**, 391.

KINSKY, S. C. & NICOLOTTI, R. A. (1977) Immunological properties of model membranes. *Ann. Rev. Biochem.*, **46**, 49.

LEDBETTER, J. & NOWINSKI, R. C. (1977) Identification of the Gross cell surface antigen associated with murine leukemia virus infected cells. *J. Virol.*, **23**, 315.

MANN, D. L. (1972) The effect of enzyme inhibitors on the solubilization of HL-A antigens with 3M KCl. *Transplantation*, **14**, 398.

NICOLOTTI, R. A., KOCHIBE, N. & KINSKY, S. C. (1976) Comparative immunogenic properties of N-substituted phosphatidylethamolamine derivatives and liposomal model membranes. *J. Immunol.*, **117**, 1898.

OLD, L. J., BOYSE, E. A. & STOCKERT, E. (1965) The G(Gross) leukemia antigen. *Cancer Res.*, **25**, 813.

OZATO, K., ZIEGLER, H. K. & HENNEY, C. S. (1978) Liposomes as model membrane systems for immune attack. I. Transfer of antigeneic determinant to lymphocyte membrane after interaction with hapten bearing liposomes. *J. Immunol.*, **121**, 1376.

SAKAI, F., GERLIER, D. & DORÉ, J. F. (1980) Association of Gross virus-associated cell-surface antigen with liposomes. *Br. J. Cancer*, **41**, 227.

SNYDER, H. W., STOCKERT, E. & FLEISSNER, E. (1977) Characterization of molecular species carrying Gross cell surface antigen. *J. Virol.*, **23**, 302.

YASUDA, T., DANCEY, G. F. & KINSKY, S. C. (1977) Immunogenicity of liposomal model membranes in mice: Dependence on phospholipid composition. *Proc. Natl Acad. Sci.*, **74**, 1234.